# Effects of nutrient loading on sediment bacterial and pathogen communities within seagrass meadows

**DOI:** 10.1002/mbo3.600

**Published:** 2018-03-09

**Authors:** Songlin Liu, Zhijian Jiang, Yiqin Deng, Yunchao Wu, Jingping Zhang, Chunyu Zhao, Delian Huang, Xiaoping Huang, Stacey M. Trevathan‐Tackett

**Affiliations:** ^1^ Key Laboratory of Tropical Marine Bio‐resources and Ecology, South China Sea Institute of Oceanology Chinese Academy of Sciences Guangzhou China; ^2^ University of Chinese Academy of Sciences Beijing China; ^3^ Key Laboratory of South China Sea Fishery Resources Exploitation & Utilization, Ministry of Agriculture South China Sea Fisheries Research Institute Chinese Academy of Fishery Sciences Guangzhou China; ^4^ School of Life and Environmental Sciences Centre for Integrative Ecology Deakin University Vic. Australia

**Keywords:** bacteria, denitrification, eutrophication, putative pathogens, seagrass meadows

## Abstract

Eutrophication can play a significant role in seagrass decline and habitat loss. Microorganisms in seagrass sediments are essential to many important ecosystem processes, including nutrient cycling and seagrass ecosystem health. However, current knowledge of the bacterial communities, both beneficial and detrimental, within seagrass meadows in response to nutrient loading is limited. We studied the response of sediment bacterial and pathogen communities to nutrient enrichment on a tropical seagrass meadow in Xincun Bay, South China Sea. The bacterial taxonomic groups across all sites were dominated by the Gammaproteobacteria and Firmicutes. Sites nearest to the nutrient source and with the highest NH_4_^+^ and PO_4_^3−^ content had approximately double the relative abundance of putative denitrifiers Vibrionales, Alteromonadales, and Pseudomonadales. Additionally, the relative abundance of potential pathogen groups, especially *Vibrio* spp. and *Pseudoalteromonas* spp., was approximately 2‐fold greater at the sites with the highest nutrient loads compared to sites further from the source. These results suggest that proximity to sources of nutrient pollution increases the occurrence of potential bacterial pathogens that could affect fishes, invertebrates and humans. This study shows that nutrient enrichment does elicit shifts in bacterial community diversity and likely their function in local biogeochemical cycling and as a potential source of infectious diseases within seagrass meadows.

## 1 INTRODUCTION

Seagrass meadows are incredibly productive ecosystems (Hemminga & Duarte, 2000) and support a high diversity of microorganisms in their sediments (Bourque, Vega‐Thurber, & Fourqurean, 2015; García‐Martínez, López‐López, Calleja, Marbà, & Duarte, 2009), due to the release of root exudates (amino acids, sugars) and oxygen into the rhizosphere (Christiaen, McDonald, Cebrian, & Ortmann, 2013; Ingemann Jensen, Kühl, Glud, Jørgensen, & Priemé, 2005; Jensen, Kühl, & Priemé, 2007). Microbes play a fundamental role in several biogeochemical cycling processes, including the oxidation of organic carbon, nitrogen fixation, nitrification, denitrification, iron cycling, and sulfate reduction within the rhizosphere and surrounding sediments (Christiaen et al., 2013; Hemminga & Duarte, 2000; Marbà, Holmer, Gacia, & Barron, 2007). Most of these processes are primarily driven by bacterial communities (Jørgensen, 1982). For example, nitrogen fixation by cyanobacteria and sulfate‐reducing bacteria can significantly contribute to seagrass nutrient requirements (Hansen, Udy, Perry, Dennison, & Lomstein, 2000; Welsh, 2000). In addition, the presence of bacterial pathogens in coastal waters and sediments are important indicators of environmental health (Bally & Garrabou, 2007; Luna et al., 2010). In fact, seagrass meadows have been shown to be involved in reducing the abundance of bacterial pathogens linked to infections and diseases in humans and marine organisms (Lamb et al., 2017).

Unfortunately, seagrass beds have been severely degraded by anthropogenic disturbances and climate change, with rapid rate of decline worldwide (~7% per year; Waycott et al., 2009). In particular, seagrass beds have been adversely affected by eutrophication, and this pressure is predicted to increase over the coming decades due to increased coastal aquaculture, coastal development and concurrent runoff (Burkholder, Tomasko, & Touchette, 2007; Ralph, Tomasko, Moore, Seddon, & Macinnis‐Ng, 2006). In addition to directly affecting seagrass health through light reduction, ammonium toxicity and water‐column nitrate inhibition (Burkholder et al., 2007), eutrophication has been shown to affect sediment bacterial metabolism and function (Howard, Perez, Lopes, & Fourqurean, 2016; López, Duarte, Vallespinós, Romero, & Alcoverro, 1998). López et al. (1998) found that the addition of inorganic nitrogen to *Posidonia oceanica* sediments significantly increased ammonification rates and bacterial exoenzymatic activities, which resulted in enhanced bacterial decomposition of seagrass‐derived carbon. Additionally, the bacterial pathogen and disease occurrence have been consistently correlated with high nutrient loads in near‐shore environments (National Research Council, 2000; Vega Thurber et al., 2014; Zaneveld et al., 2016). However, studies that investigate the effect of nutrient loading on sediment bacterial community structure within seagrass meadows are otherwise rare (Guevara, Ikenaga, Dean, Pisani, & Boyer, 2014). Filling this gap could help us understand the factors driving nutrient cycling and the potential for disease outbreaks resulting from nutrient loading within seagrass meadows.

In this study, we used Next‐generation sequencing to answer the question: To what extent does nutrient enrichment affect the sediment bacterial community structure, including putative pathogens, in a seagrass meadow? We sampled sediments from mixed seagrass communities, with increasing distance from nutrient loads coming from fish cages in Xincun Bay, South China Sea (Liu et al., 2016; Zhang, Huang, & Jiang, 2014). Our previous research has indicated that high nutrient levels in Xincun Bay stimulate the seagrass, macroalgal and epiphytic biomass production, resulting in higher organic carbon availability for bacteria or contribution to sediment carbon stocks (Liu et al., 2016, 2017). We hypothesized that higher nutrient loads from the fish cages will alter the bacterial community structure and bacterial pathogens in seagrass sediments. The results of this study on how microbial community composition changes under eutrophication scenarios will help clarify the reciprocal relationships of microbes and the biogeochemical environment in degraded seagrass meadows, and increase our understanding of the microbial ecology in these ecologically and socioeconomically important ecosystems.

## 2 MATERIALS AND METHODS

### 2.1 Study area and sampling sites

**Figure 1:**
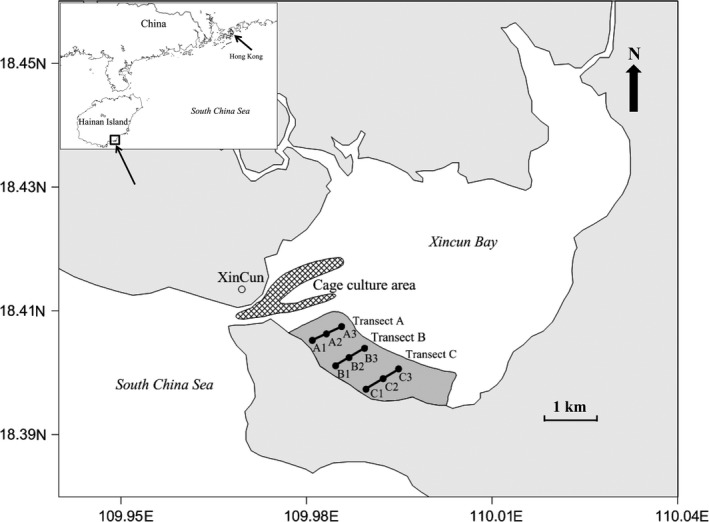
Sampling sites in Xincun Bay at Hainan Island in the South China Sea, which were divided by distance from the cage aquaculture (transects A, B and C) and distance from the shore (stations 1, 2 and 3)

The study was performed at Xincun Bay (18°24′34′′N–18°24′42′′N, 109°57′42′′E–109°57′58′′E), located in southeastern Hainan Island, South China Sea (Figure 1). In recent years, cage aquaculture, located near the entrance of the bay, has developed rapidly, and currently includes more than 450 floating cage units (Zhang et al., 2014). At the south‐eastern part of the fish farm, shallow‐water seagrass meadows dominated by *Thalassia hemprichii* (Ehrenb. ex Solms) Asch. and *Enhalus acoroides* (L.f.) Royle occupy an area of approximately 200 ha (Huang et al., 2006). Three transects (A, B, C) were selected according to the distance to fish farm in the seagrass bed and represented a nutrient load gradient (Liu et al., 2016). The first transect was 500 m from the fish farm, with the other transects ~800 m apart from each other. For each transect, independent samples were taken at sampling stations at 50, 400 and 750 m from the shore (stations 1, 2 and 3, respectively). *E acoroides* was not found at stations B1, C1 and C2, and *T*. *hemprichii* was not found at A2. Stations A1, A3, B2, B3, and C3 included both seagrass species (Figure 1). Each sample was annotated according to transect, station and seagrass species, for example, *T*. *hemprichii* in A1 can be represented as A1.T.

### 2.2 Sample collection

During low tide (~ 10 cm depth), 1 L of seawater above the sediment was sampled for nutrient analysis. Additionally, a surface sediment sample (0–3 cm) was collected in both *T. hemprichii* and *E. acoroides* meadows at each site, using a sterile 10 cm diameter sampling core. Each sediment sample was divided into two subsamples, one subsample was frozen at −20°C for sediment organic carbon (SOC) and sediment total nitrogen (TN) analysis. The other subsample was preserved at −80°C until extraction of genomic DNA.

### 2.3 Sample preparation and analysis

#### 2.3.1 Seawater nutrients, SOC and sediment TN analysis

The seawater was filtered onto precombusted GF/F filters (Whatman, 450°C, 3 hr). The filtered seawater was analyzed for NO_3_^−^–N, NO_2_^−^–N, NH_4_^+^–N, and PO_4_^3−^, using an AQ‐2 Automated Discrete Analyzer (Seal Analytical Inc.). The sum of NO_3_^−^–N, NO_2_^—^N, and NH_4_^+^–N represents the total concentration of dissolved inorganic nitrogen (DIN). The frozen (−20°C) sediment samples were freeze‐dried and composited by plot in order to make the samples homogeneous. The composite samples were sieved through a 500‐μm screen to remove coarse sediment and detrital materials. The samples were then ground and homogenized with a mortar and pestle. Sediment was acidified with 1 mol/L HCl overnight at room temperature to remove carbonate. Acidified sediments were washed with distilled water and dried at 40°C in an oven. SOC (acidified) and sediment TN (unacidified) was determined, using an elemental analyzer (Vario EL, Elemental Analyser systeme GmbH, Germany).

#### 2.3.2 DNA extraction, PCR amplification and Illumina sequencing

DNA was extracted from 0.5 g of sediment (wet weight), using an EZNA^®^ Soil DNA Kit (Omega Bio‐Tek Inc., Norcross, GA, USA), according to the manufacturer's instructions. The quality and quantity of the extracted DNA were verified by measuring the OD260/280 (≈1.8) with a Nanodrop 2000 spectrophotometer and agarose gel electrophoresis, respectively. Primer set 515F/806R was used to amplify the V4 region of 16S rRNA gene (Mckirdy et al., 2010). Sequencing was performed on an Illumina MiSeq platform at Novogene Genomics Technology Co. Ltd, Beijing, China.

#### 2.3.3 Sequence analyses, OTU clustering and bioinformatics analysis

Sequences were analyzed, using the QIIME version 1.9.1 pipeline (Caporaso et al., 2010b). Raw sequences were demultiplexed and quality filtered, using the default parameters in QIIME. Sequences were then clustered into operational taxonomic units (OTUs), which was defined as >97% 16S rRNA gene sequence similarity, using UCLUST (Edgar, 2010) with the open reference clustering protocol. The resulting representative sequences set were aligned, using PyNAST (Caporaso et al., 2010a) and given a taxonomic classification, using RDP (Wang, Garrity, Tiedje, & Cole, 2007), retrained with the Greengenes version 13.5 (McDonald et al., 2012). The resulting OTU table was used to determine the Chao 1, Shannon, and rarefaction diversity indices, using QIIME. METAGENassist was used for Weighted Unifrac analysis (Arndt et al., 2012). In addition, we reviewed papers containing information pertinent to the putative pathogens (genus level) that can cause fish and invertebrate disease or death or cause human illness through the consumption of seafood or skin contact. A list of putative pathogen OTUs (total abundance of all samples >0.1) was compiled for further statistical analysis.

### 2.4 Statistical analysis

The raw data were log‐ or exponent‐transformed in order to fulfill the assumptions of homogeneity and normality in cases where these assumptions were not met. Since the effect of nutrient loading was the treatment we were primarily interested in testing, we ran a preliminary one‐way analysis of variance (ANOVA) to examine the effect of distance to shore on seawater nutrients (DIN, NO_3_^−^–N, NO_2_^−^–N, NH_4_^+^–N and PO_4_^3−^), SOC, sediment TN, and sediment *C*/*N*. Distance from the shore was not a significant treatment affecting the response variables (*p* > .05), and thus were pooled for further statistical tests. A one‐way ANOVA tested the effect of transect on seawater nutrients, while a two‐way ANOVA analyzed the effects of transect and seagrass species on the SOC, sediment TN and sediment *C*/*N*. Whenever significant differences were observed from an ANOVA, a Tukey's post hoc test was run to identify the significantly different components of dependent variables.

The weighted UNIFRAC resemblance matrix (Table S1) was used for the bacterial community analyses, while the filtered putative pathogen counts were used for pathogen community analyses. For both datasets, a preliminary one‐way permutational multivariate ANOVA (PERMANOVA) showed that distance to shore was not a significant treatment (Pseudo‐*F* = 0.8236, *P‐perm* = 0.569). A two‐way PERMANOVA was subsequently performed to determine the statistically significant differences among the transects and seagrass species. All main and interaction effects with α < 0.05 were considered statistically significant. A similarity percentage analysis (SIMPER) was used to identify the bacterial and pathogenic taxa driving the differences in treatments. The relationships between environmental parameters (seawater nutrients, SOC and sediment TN) and the entire bacterial community relative abundance data were evaluated by distance‐based redundancy analyses (db‐RDA) (Legendre & Anderson, 1999). The above‐mentioned statistical analyses were performed with PRIMER 6 & PERMANOVA+ (Clarke & Gorley, 2006) and IBM SPSS Statistics 19.0 software, respectively.

## 3 RESULTS

### 3.1 Variations of seawater nutrient and sediment parameters

The DIN and PO_4_^3−^ concentrations of the seawater ranged from 4.57 to 15.29 μmol·L^−1^ and 0.32 to 0.97 μmol·L^−1^, respectively (Table 1). The DIN species was dominated by NH_4_^+^. Significantly higher DIN (*F* = 75.71, *p* = .000), NH_4_^+^ (*F* = 135.72, *p* = .000) and PO_4_^3−^ (*F* = 14.73, *p* = .005) concentrations were found in transect A compared to the other transects (Tukey's post hoc test). The SOC, sediment TN, and sediment *C*/*N* ranged from 0.09% to 0.73%, 0.015% to 0.053% and 7.40 to 16.07, respectively, with the highest SOC, TN, and *C*/*N* found at site A1.T (Table 1). Additionally, the sediment *C*/*N* ratios were not observed to be significantly different among transects (*F* = 2.08, *p* = .188); however, there was significantly higher SOC (*F* = 8.936, *p* = .009) and sediment TN (*F* = 16.53, *p* = .001) content observed in transect A compared to the other transects (Tukey's post hoc test). SOC, sediment TN, and sediment *C*/*N* were not affected by the seagrass species and the interactions of transect and seagrass species (*p *>* *.05).

**Table 1:** Seawater nutrients and sediment elemental content among all the sampling stations

Parameters		Stations								
		A1	A2	A3	B1	B2	B3	C1	C2	C3
DIN (μmol·L^−1^)		12.86	15.29	12.71	5.62	5.16	6.41	4.87	5.51	4.57
NH_4_^+^ (μmol·L^−1^)		10.68	12.46	11.38	4.07	3.75	4.88	3.56	3.86	3.09
NO_3_^−^ (μmol·L^−1^)		1.94	2.67	1.12	1.35	1.22	1.48	1.14	1.48	1.29
NO_2_^−^ (μmol·L^−1^)		0.24	0.16	0.21	0.19	0.19	0.05	0.16	0.17	0.19
PO_4_^3−^ (μmol·L^−1^)		0.97	0.68	0.73	0.42	0.47	0.32	0.45	0.40	0.35
*Thalassia hemprichii*	SOC (%)	0.73		0.33	0.16	0.14	0.17	0.13	0.09	0.14
	Sediment TN (%)	0.053		0.039	0.025	0.021	0.018	0.015	0.01	0.013
	Sediment *C*/*N*	16.07		9.87	7.47	7.78	11.02	10.11	10.5	12.56
*Enhalus acoroides*	SOC (%)	0.26	0.22	0.32		0.1	0.16			0.15
	Sediment TN (%)	0.041	0.029	0.035		0.015	0.025			0.021
	Sediment *C*/*N*	7.40	8.85	10.67		7.78	7.47			8.33

### 3.2 Total bacterial community structure

Sequencing of the sediment microbial communities, using the 16S rRNA gene resulted in more than 388 k sequences and 2.5 k OTUs from all the sampling sites. On average, each sample had ~ 28,000 sequences and ~1,850 OTUs (Table S2). The α‐diversity indices (Chao1, Shannon, and rarefaction) indicated that there was lower diversity in transect A compared to the other transects (Table S2).

**Figure 2:**
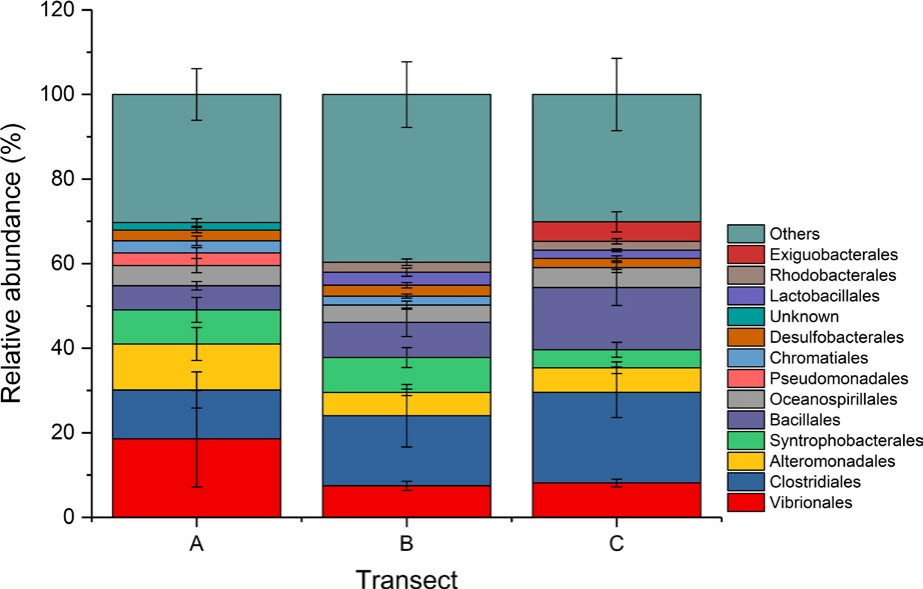
Microbial community composition of the top 10 most abundant orders averaged over each transect. Values show means and 1 standard error (*n* = 4–5). Relative abundances at each site are provided in Figure S1

Though there was no significant effect on bacterial community structure associated with seagrass species (Pseudo‐ *F* = 2.1021, *P‐perm* = 0.115) or the transect *x* species interaction (Pseudo‐ *F* = 1.988, *P‐perm* = 0.129), the effect of transect location on the bacterial communities was marginally significant (Pseudo‐ *F* = 2.3613, *P‐perm* = 0.079). SIMPER analysis showed that the 10 most abundant orders accounted for more than 60% of the dissimilarity between transects and was dominated by the Gammaproteobacteria and Firmicutes (Figure 2 and Figure S1, Table S3). In transect A, the average relative abundance of Vibrionales, Alteromonadales, and Pseudomonadales, belonging to denitrifying bacteria groups, were up to twofold higher compared to the other two transects, while the Bacillales and Clostridiales were higher in transects B and C (Figure 2). Furthermore, the relative abundance of Clostridiales, Bacillales, and Exiguobacterales increased from transect A to transect C, while that of Pseudomonadales showed the opposite trend (Figure 2).

**Figure 3:**
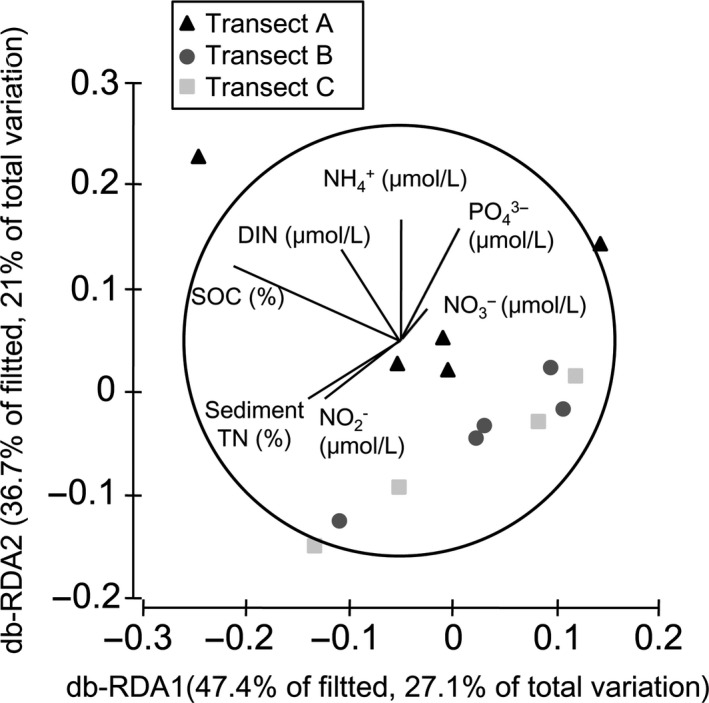
Distance‐based Redundancy Analysis (db‐RDA) ordination of microbial community data (Weighted UNIFRAC resemblance matrix calculated from relative abundance data) fitted to environmental variables. The plot represents a db‐RDA ordination based upon the Bray–Curtis distance of all the sampling sites. Correlations can be found in Table 2

The db‐RDA analyses of the 16S rRNA gene data explained 48.1% of the variation in the first two axes (Figure 3). The db‐RDA analyses confirmed the clear separation of transects according to above‐mentioned environmental factors, although much of this difference looks to be driven by one sample (A1.T). Axis 2 (db‐RDA2) separated the sites furthest from fish farms sites (including transect B and C) from the nearer sites (transect A) in Xincun Bay. The transect profiles reflected an apparent shift in SOC and nutrient concentrations between A and B/C (Figure 3). There were strong negative correlations between SOC and db‐RDA1 (*r* = −.755; Table 2), and strong positive correlations between NH_4_^+^ (*r* = .532), PO_4_^3−^ (*r* = .52) and db‐RDA2, respectively (Table 2).

**Table 2:** Multiple partial correlations between Distance‐based Redundancy Analysis (db‐RDA) coordinate axes and environmental variables

Variable	db‐RDA1	db‐RDA2
SOC (%)	−0.755	0.334
Sediment TN (%)	−0.342	−0.263
DIN (μmol/L)	−0.264	0.418
NH_4_^+^ (μmol/L)	0.022	0.532
NO_3_^−^ (μmol/L)	0.112	0.148
NO_2_^−^ (μmol/L)	−0.398	−0.262
PO_4_^3−^ (μmol/L)	0.269	0.52

### 3.3 Putative pathogens

**Figure 4:**
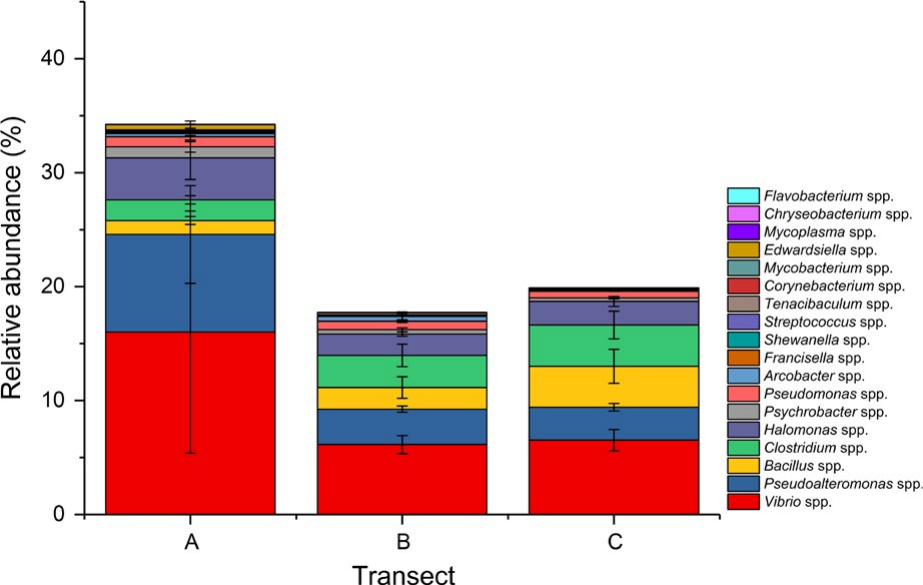
Presence of putative pathogens at the genus level averaged over the three transects. Values show means and 1 standard error (*n* = 4–5). Relative abundances at each site are provided in Figure S2

There were 18 potentially opportunistic, putative pathogens found in the seagrass sediments in this study, including those associated with human, fish, invertebrate, and mammal diseases (Table 3). The putative pathogens (at genus level) accounted for about 24.25% of total bacterial community among all the transects. In general, these taxa presented in higher relative abundances in transect A, particularly at the *T. hemprichii* stations in transect A, than other all other stations (Figure 4 and Figure S2). Although the PERMANOVA indicated that the overall bacterial community composition was not significantly different among the three transects, total pathogenic relative abundance in transect A was 1.8 times that of the other two transects. The average dissimilarities of transect A versus transect B and transect A versus transect C were twice as that of transect B versus transect C (Table S4). Abundance differences in *Vibrio* spp. and *Pseudoalteromonas* spp. contributed to more than 60% of the dissimilarities between transect A and the other transects (Table S4). Moreover, the relative abundances of *Vibrio* spp. and *Pseudoalteromonas* spp. were both more than twofold higher in transect A compared to transects B and C.

**Table 3:** A list of putative pathogens of human, fishes, and invertebrates identified in this study

Taxon	Infectious organisms	References
*Arcobacter* spp.	Human	Collado, Inza, Guarro, and Figueras (2008)
*Bacillus* spp.	Fishes and invertebrates	Webster (2007) and Austin, Austin, Austin, and Austin (2012)
*Chryseobacterium* spp.	Fishes	Austin et al. (2012)
*Clostridium* spp.	Human	Gorbach and Thadepalli (1975)
*Corynebacterium* spp.	Human	Roux et al. (2004)
*Edwardsiella* spp.	Human and Fishes	Bullock and Herman (1985) and Obasohan, Agbonlahor, and Obano (2010)
*Flavobacterium* spp.	Fishes	Farkas (1985)
*Francisella* spp.	Fishes	Mauel, Soto, Moralis, and Hawke (2007)
*Halomonas* spp.	Human	Stevens, Hamilton, Johnson, Kim, and Lee (2009)
*Mycobacterium* spp.	Human and Fishes	Primm, Lucero, and Falkinham (2004) and Watral and Kent (2007)
*Mycoplasma* spp.	Human and invertebrates	Paillard, Le Roux, and Borrego (2004) and Waites, Katz, and Schelonka (2005)
*Pseudoalteromonas* spp.	Invertebrates	Chistoserdov, Gubbala, Smolowitz, Mirasol, and Hsu (2005)
*Pseudomonas* spp.	Human and invertebrates	Gilardi (1972) and Webster (2007)
*Psychrobacter* spp.	Human	Bowman (2006)
*Shewanella* spp.	Human and invertebrates	Li et al. (2010) and Janda (2014)
*Streptococcus* spp.	Fishes	Baeck, Kim, Gomez, and Park (2006)
*Tenacibaculum* spp.	Fishes	Austin et al. (2012)
*Vibrio* spp.	Human, fishes and invertebrates	Colwell and Grimes (1984), Janda, Powers, Bryant, and Abbott (1988) and Vaseeharan and Ramasamy (2003)

## 4 DISCUSSION

The objective of this work was to study the effects of aquaculture‐associated nutrient loading on the bacterial community structure in seagrass sediments, including the presence of putative pathogens. Overall the nutrient concentrations of this study were generally higher than other nutrient‐impacted seagrass beds (Apostolaki, Holmer, Marbà, & Karakassis, 2010; Guevara et al., 2014), which can mainly be attributed to the large amount of floating fish cage units in a nearly closed‐system Xincun Bay. On a local scale, the nutrient concentrations in the seawater were highest in the areas closest to the fish farming area, which was similar to that observed in previous studies carried out in Xincun Bay (Zhang et al., 2014). Liu et al. (2016) revealed that the SOC source in seagrass meadows of Xincun Bay was of marine autochthonous origin, with more algal organic carbon contribution in the high nutrient areas. This indicated that the aquaculture‐induced nutrient loading enhanced more labile organic carbon inputs, and the import of aquaculture‐sourced particulate organic matter to seagrass meadows was negligible. Therefore, the results of this study provided evidence that eutrophic inputs, including labile organic carbon inputs, could be shifting the sediment bacterial community toward functional groups that can utilize the excess nutrients. The elevated denitrifying communities and putative pathogens in the high nutrient‐loaded area (transect A) coupled with the reduced seawater nutrient load and shift in microbial communities in transects B and C also suggest that the seagrass plants themselves and their associated microbial communities could be playing a role in minimizing the influence and spread of nutrients and pathogens to nearby areas, potentially through metabolic and biogeochemical cycling and filtration.

### 4.1 Characteristics of bacterial and pathogen community structure

In this study, high bacterial community diversity (Shannon index of this study: 3.72–8.72; other studies: 3.71–4.00; Ikenaga, Guevara, Dean, Pisani, & Boyer, 2010; Guevara et al., 2014) could contribute to the maintenance, function and stability of the environment, and thus improve the resilience of ecosystems suffering from human disturbance (Parnell, Crowl, Weimer, & Pfrender, 2009). Consistent with a previous study in Xincun Bay (Jiang et al., 2015), the Gammaproteobacteria and Firmicutes were the dominant bacterial taxonomic groups in the surface sediments. Fish farming typically generates a large amount of feces that are abundant in Firmicutes (Wu et al., 2010), which explains the relative high abundance of Firmicutes in this study. However, this study showed some dissimilar results compared with other regional seagrass meadows’ studies that showed sediments primarily dominated by Deltaproteobacteria and Bacteroidetes (García‐Martínez et al., 2009; Guevara et al., 2014; Ikenaga et al., 2010). Most Deltaproteobacteria are sulfate reducers and occur in anaerobic conditions (de Moraes, Franco, Pellizari, & Sumida, 2014; López‐García et al., 2003). Bacteroidetes were associated with substrates rich in organic carbon (Fierer, Bradford, & Jackson, 2007). The sandy substrate and the low sediment organic matter in the seagrass beds of Xincun Bay (Liu et al., 2016, 2017) likely promote deeper oxygen penetration, which could reduce the microbially mediated sulfate reduction metabolic pathways in sediments (Bourque et al., 2015). This possibly explains the relatively low abundances of the Deltaproteobacteria and Bacteroidetes in this study.

Along similar trends, the pathogenic groups were mainly assigned to Gammaproteobacteria and Firmicutes. Our sequences aligned with 18 of 42 potentially pathogenic genera described in Lamb et al. (2017). Lamb et al. (2017) reported that the relative abundance of potentially pathogenic genera was less than 1% in seawater in seagrass meadows at Spermonde Archipelago, Indonesia. This concentration was much lower than the sediment pathogenic relative abundance (24%) in this study. It has been previously shown that pathogen abundance can be up to 100‐fold greater in sediment when compared with the water column (Ghaderpour et al., 2014; Perkins et al., 2014), and this may indicate that the sediment in seagrass meadows may be a sink for pathogens in the water column that move across a seagrass canopy. However, more work needs to be done to link the high relative abundances of putative pathogenic genera to the actual pathogenic species or strain as well as to actual cell counts in order to fully understand the risks of pathogenic accumulation to human and ecosystem health.

### 4.2 Responses of bacterial and pathogenic community structure to nutrient load

We found preliminary evidence that sediment bacterial community structure could be influenced by the proximity to point source nutrients loads. The db‐RDA2 axis indicated high nutrient load sampling stations were separated from the other sampling stations, although the PERMANOVA results indicated no statistical difference. These disparate results were likely due to the variability in seagrass meadow diversity as well as low replication sampling within a transect. However, despite the low statistical power, the db‐RDA analysis indicated that NH_4_^+^ and PO_4_^3−^ contents were important drivers of the microbial community structure between transect A and transects B and C, that is, proximity to nutrient source. It was previously shown that microbial communities of salt marsh sediments can also be stabilized in response to nutrient loading (Bowen et al., 2011; Kearns et al., 2016). In this study, it is possible that NH_4_^+^ and PO_4_^3−^, typically the limiting factors for heterotrophic bacteria production (Kirchman, 1994; Thingstad, Zweifel, & Rassoulzadegan, 1998; Wheeler & Kirchman, 1986), are directly synthesized into bacterial biomass and promoting bacterial growth. Furthermore, higher eutrophic conditions can also lead to higher labile organic carbon inputs, which was apparent in transect A (Liu et al., 2016). Elevated nutrient load in combination with labile organic carbon availability favors the fast‐growing *r*‐strategist microbes (Fernandes, Kirchman, Michotey, Bonin, & LokaBharathi, 2014; Pinhassi & Berman, 2003; Trevathan‐Tackett et al., 2017) and is likely linked to the higher relative abundances of Gammaproteobacteria, like Vibrionales, Alteromonadales, and Pseudomonadales, in transect A.

In general, the Gammaproteobacteria represent abundant denitrifying communities in marine sediments (Bhatt, Zhao, Monteil‐Rivera, & Hawari, 2005). Here, the results indicate that the carbon‐limited conditions of this study area, indicated by low sediment *C*/*N* ratios and SOC content (this study: average value was 0.22%; global data: average value was 1.8%; Kennedy et al., 2010) and high proportion of microbial biomass carbon in SOC (previous study in Xincun Bay: average value was 15.74%; Liu et al., 2016), would favor denitrification processes (Burgin & Hamilton, 2007). The process of nitrate being converted to N_2_ would be enhanced in high nutrient areas due to the potential high denitrification activity. This may be benefiting nearby areas by reducing nitrate loads in areas closest to the fish farming area. Conversely, at the relative low nutrient concentration areas, there were increases in Firmicutes, including members of the Bacillales, Clostridiales, and Exiguobacterales. Liu et al. (2016, 2017) have found that bacterial biomass and labile organic carbon decreased with decreasing nutrient concentrations in Xincun Bay. The Firmicutes is relative stable group of bacteria under various substrate availability conditions (Kampmann et al., 2012), therefore the increase in their relative abundance at the lower nutrient transects might be due to the relative shifts in other bacterial groups.

The pathogenic population was about twofold greater in high nutrient load areas than that of the relatively lower nutrient areas. This was consistent with previous theoretical and empirical studies that nutrient enrichment often enhances pathogen abundance due to high resource availability, including abundant labile carbon substrates (Johnson et al., 2010; Lafferty & Holt, 2003; Liu et al., 2016; McKenzie & Townsend, 2007). Previous work on the causative agent of cholera (*Vibrio cholerae*) provided a good example of how a pathogen can be affected by nutrient load and related marine plankton bloom (McKenzie & Townsend, 2007; Vezzulli, Pruzzo, Huq, & Colwell, 2010). Secondly, high nutrient loads have led to a decline in seagrass biomass and cover in this study area (Liu et al., 2016), which could reduce their ‘pathogen filtering’ abilities within the seagrass meadows (Lamb et al., 2017). Thirdly, the elevated nutrient load could also increase pathogen fitness and virulence or change host susceptibility (Johnson et al., 2010; McKenzie & Townsend, 2007). In this study, *Vibrio* spp. and *Pseudoalteromonas* spp. were mainly responsible for the differences in pathogen abundances under different nutrient conditions. The nutrient load induced by fish farms could heighten the prevalence risk of infectious diseases in natural fishery resources as well as aquaculture organisms in Xincun Bay (Iwamoto, Ayers, Mahon, & Swerdlow, 2010; Johnson et al., 2010; Oetama et al., 2016). Additionally, there are more than 12 species of *Vibrio* known as human pathogens (Blazer, 1988), resulting in disease as a consequence of toxin intake (Martinez‐Urtaza et al., 2012; Oetama et al., 2016). Furthermore, the sediment pathogens can be grazed associated with sediment organic matter at the bottom, by meiofauna, worms, prawns, cockles, and demersal fishes, sequentially may move up the food chain and reach humans (Ghaderpour et al., 2014).

## 5 CONCLUSION

We found that higher nutrient load into a seagrass meadow elevated the relative abundance of denitrifying communities as well as potential pathogen groups. Changes of sediment bacteria and pathogen relative abundance not only should be attributed to nutrient enrichment, but also associated labile organic carbon. On one hand, these findings imply that high nutrient enrichment enhanced the potential denitrification activity, yet present a risk of infectious diseases in nature. What is unknown, however, is how the bacterial and pathogenic activities change in response to nutrient loading. As a matter for future research, we recommend meta‐transcriptomic and metabolomics analyses of seagrass sediment microbial communities to evaluate functional gene expression associated with nutrient cycling and virulence products among different nutrient load levels.

## CONFLICT OF INTEREST

None declared.
