# Supplementary material for: Effects of nutrient loading on sediment bacterial and pathogen communities within seagrass meadows
Source: Microbiologyopen. 2018 Mar 9;7(5):e00600. doi: 10.1002/mbo3.600 (PMC6182560; doi:10.1002/mbo3.600)
Supplement: Supplementary file 2 [file MBO3-7-e00600-s002.doc]

***Supplementary information***

Table S1. Summary of weighted Unifrac distance matrix between all the sampling sites.

| Sites | A1.T | A3.T | A1.E | A2.E | A3.E | B1.T | B2.T | B3.T | B2.E | B3.E | C1.T | C2.T | C3.T | C3.E |
| --- | --- | --- | --- | --- | --- | --- | --- | --- | --- | --- | --- | --- | --- | --- |
| A1.T |  |  |  |  |  |  |  |  |  |  |  |  |  |  |
| A3.T | 0.296187 |  |  |  |  |  |  |  |  |  |  |  |  |  |
| A1.E | 0.374592 | 0.2933612 |  |  |  |  |  |  |  |  |  |  |  |  |
| A2.E | 0.3893739 | 0.2956885 | 0.14308 |  |  |  |  |  |  |  |  |  |  |  |
| A3.E | 0.3997913 | 0.3158356 | 0.1596643 | 0.110872 |  |  |  |  |  |  |  |  |  |  |
| B1.T | 0.4176987 | 0.2047378 | 0.3648785 | 0.3582274 | 0.3567218 |  |  |  |  |  |  |  |  |  |
| B2.T | 0.4815305 | 0.3983008 | 0.1994583 | 0.2351875 | 0.2473585 | 0.4248623 |  |  |  |  |  |  |  |  |
| B3.T | 0.4377627 | 0.3511573 | 0.1459688 | 0.1977302 | 0.2132245 | 0.3963742 | 0.1278444 |  |  |  |  |  |  |  |
| B2.E | 0.4549745 | 0.3697161 | 0.1800808 | 0.1742655 | 0.1825028 | 0.3729 | 0.1722225 | 0.1619001 |  |  |  |  |  |  |
| B3.E | 0.3858043 | 0.1995362 | 0.2712151 | 0.2591814 | 0.2491841 | 0.1695205 | 0.33708 | 0.2985479 | 0.286128 |  |  |  |  |  |
| C1.T | 0.4590848 | 0.3749934 | 0.1757225 | 0.2288419 | 0.2413147 | 0.4163078 | 0.1225244 | 0.0819924 | 0.1876568 | 0.3176366 |  |  |  |  |
| C2.T | 0.4341164 | 0.2281041 | 0.3564851 | 0.338135 | 0.3333056 | 0.1160161 | 0.4061558 | 0.3814955 | 0.3429104 | 0.1566164 | 0.3972637 |  |  |  |
| C3.T | 0.3846054 | 0.179448 | 0.338373 | 0.3300853 | 0.3372245 | 0.0924336 | 0.4003779 | 0.3695119 | 0.356508 | 0.135563 | 0.3879857 | 0.1075463 |  |  |
| C3.E | 0.3767154 | 0.2061234 | 0.2783008 | 0.2658492 | 0.2703472 | 0.1907885 | 0.3516051 | 0.3221476 | 0.3049642 | 0.0940454 | 0.3421452 | 0.1807476 | 0.1610789 |  |

Table S2. Summary of Illumina sequencing of each site sediment.

| Sites | No. of sequences | No. of OTUs | α-diversity indices | | |
| --- | --- | --- | --- | --- | --- |
|  |  |  | Chao1 | Shannon | Rarefaction |
| A1.T | 38483 | 1141 | 1141.58 | 3.72 | 558 |
| A3.T | 36242 | 1584 | 1368.02 | 5.67 | 788 |
| A1.E | 32037 | 2317 | 2230.90 | 8.22 | 1471 |
| A2.E | 33669 | 2518 | 2133.84 | 8.35 | 1564 |
| A3.E | 22475 | 2120 | 2051.34 | 8.11 | 1470 |
| B1.T | 34741 | 1496 | 1304.37 | 6.30 | 797 |
| B2.T | 34614 | 2620 | 2263.39 | 8.58 | 1677 |
| B3.T | 32421 | 2642 | 2315.69 | 8.62 | 1676 |
| B2.E | 10647 | 1545 | 2235.10 | 8.40 | 1545 |
| B3.E | 13379 | 1106 | 1103.77 | 6.75 | 880 |
| C1.T | 31397 | 2676 | 2766.56 | 8.72 | 1823 |
| C2.T | 21476 | 1397 | 1617.53 | 6.48 | 972 |
| C3.T | 19909 | 1188 | 1338.47 | 6.26 | 1018 |
| C3.E | 27045 | 1523 | 1512.41 | 6.59 | 949 |

Diversity indices were calculated by using an equal number of sequences (10,640) subsampled 10 times from original libraries.

Table S3. Results of the order-level SIMPER analysis giving the dissimilarities of total bacterial communities between transects. The top 10 orders that contribute most to the dissimilarity are listed. SD represents standard deviation.

|  | Average abundance of transect A | Average Abundance of transect B | Average Dissimilarity | Dissimilarity/SD | Contribution % | Cumulative contribution % |
| --- | --- | --- | --- | --- | --- | --- |
| **Transect A *vs.* Transect B**  **(average dissimilarity 43.72)** |  |  |  |  |  |  |
| Vibrionales | 0.19 | 0.07 | 7.41 | 0.7 | 16.95 | 16.95 |
| Clostridiales | 0.12 | 0.17 | 6.25 | 0.96 | 14.3 | 31.25 |
| Alteromonadales | 0.11 | 0.06 | 3.13 | 0.84 | 7.17 | 38.42 |
| Syntrophobacterales | 0.08 | 0.08 | 3.06 | 1.35 | 7 | 45.42 |
| Bacillales | 0.06 | 0.08 | 3.01 | 1.31 | 6.88 | 52.3 |
| Oceanospirillales | 0.05 | 0.04 | 1.28 | 0.83 | 2.93 | 55.23 |
| Chromatiales | 0.03 | 0.02 | 1.07 | 1.56 | 2.45 | 57.68 |
| Pseudomonadales | 0.03 | 0.01 | 1.04 | 0.89 | 2.38 | 60.06 |
| Lactobacillales | 0.02 | 0.03 | 0.8 | 0.85 | 1.83 | 61.9 |
| Other | 0.02 | 0.02 | 0.75 | 1.34 | 1.71 | 63.61 |
| **Transect A *vs.* Transect C**  **(average dissimilarity 46.83)** |  |  |  |  |  |  |
| Vibrionales | 0.19 | 0.08 | 7.34 | 0.7 | 15.68 | 15.68 |
| Clostridiales | 0.12 | 0.21 | 6.95 | 1.46 | 14.84 | 30.52 |
| Bacillales | 0.06 | 0.15 | 5.28 | 1.93 | 11.27 | 41.79 |
| Alteromonadales | 0.11 | 0.06 | 3.23 | 0.89 | 6.9 | 48.69 |
| Syntrophobacterales | 0.08 | 0.04 | 3.16 | 1.41 | 6.75 | 55.45 |
| Exiguobacterales | 0.01 | 0.05 | 2.03 | 1.03 | 4.33 | 59.78 |
| Oceanospirillales | 0.05 | 0.05 | 1.43 | 1.01 | 3.05 | 62.82 |
| Chromatiales | 0.03 | 0.01 | 1.15 | 1.32 | 2.46 | 65.28 |
| Pseudomonadales | 0.03 | 0.01 | 1.06 | 0.84 | 2.26 | 67.54 |
| Aeromonadales | 0.01 | 0.02 | 0.87 | 0.9 | 1.86 | 69.4 |
| **Transect B *vs.* Transect C**  **(average dissimilarity 38.41)** |  |  |  |  |  |  |
| Clostridiales | 0.17 | 0.21 | 7.78 | 1.47 | 20.26 | 20.26 |
| Bacillales | 0.08 | 0.15 | 4.84 | 1.37 | 12.6 | 32.85 |
| Syntrophobacterales | 0.08 | 0.04 | 2.8 | 1.37 | 7.3 | 40.15 |
| Exiguobacterales | 0.02 | 0.05 | 2.07 | 1.14 | 5.38 | 45.53 |
| Alteromonadales | 0.06 | 0.06 | 1.16 | 1.44 | 3.03 | 48.56 |
| Vibrionales | 0.07 | 0.08 | 1.08 | 1.23 | 2.8 | 51.36 |
| Oceanospirillales | 0.04 | 0.05 | 1.03 | 0.99 | 2.68 | 54.04 |
| Aeromonadales | 0 | 0.02 | 0.73 | 0.62 | 1.91 | 55.95 |
| Lactobacillales | 0.03 | 0.02 | 0.73 | 0.79 | 1.9 | 57.85 |
| Rhodobacterales | 0.02 | 0.02 | 0.73 | 1.11 | 1.89 | 59.74 |

**Fig. S1**

**Fig. S2**

Table S4. Results of the SIMPER analysis giving the dissimilarities of pathogen communities between transects. Five orders which contribute most to the dissimilarity are listed. SD represents standard deviation.

|  | Average abundance of transect A | Average abundance of transect B | Average dissimilarity | Dissimilarity/SD | Contribution % | Cumulative contribution % |
| --- | --- | --- | --- | --- | --- | --- |
| **Transect A *vs.* Transect B (average dissimilarity 49.23)** |  |  |  |  |  |  |
| *Vibrio spp.* | 0.16 | 0.06 | 18.27 | 0.83 | 37.12 | 37.12 |
| *Pseudoalteromonas spp.* | 0.09 | 0.03 | 13.18 | 0.98 | 26.77 | 63.89 |
| *Halomonas spp.* | 0.04 | 0.02 | 5.45 | 0.59 | 11.08 | 74.97 |
| *Clostridium spp.* | 0.02 | 0.03 | 3.51 | 1.11 | 7.13 | 82.1 |
| *Bacillus spp.* | 0.01 | 0.02 | 2.8 | 0.87 | 5.68 | 87.78 |
|  |  |  |  |  |  |  |
| **Transect A *vs.* Transect C (average dissimilarity 50.35)** |  |  |  |  |  |  |
| *Vibrio spp.* | 0.16 | 0.07 | 17.61 | 0.83 | 34.98 | 34.98 |
| *Pseudoalteromonas spp.* | 0.09 | 0.03 | 12.75 | 0.99 | 25.33 | 60.31 |
| *Halomonas spp.* | 0.04 | 0.02 | 5.41 | 0.64 | 10.75 | 71.06 |
| *Bacillus spp.* | 0.01 | 0.04 | 5.37 | 1.05 | 10.67 | 81.74 |
| *Clostridium spp.* | 0.02 | 0.04 | 4.19 | 1.09 | 8.33 | 90.06 |
|  |  |  |  |  |  |  |
| **Transect B *vs.* Transect C (average dissimilarity 25.55)** |  |  |  |  |  |  |
| *Bacillus spp.* | 0.02 | 0.04 | 7.37 | 1.25 | 28.86 | 28.86 |
| *Clostridium spp.* | 0.03 | 0.04 | 6.41 | 1.49 | 25.1 | 53.96 |
| *Vibrio spp.* | 0.06 | 0.07 | 5.17 | 1.17 | 20.25 | 74.21 |
| *Halomonas spp.* | 0.02 | 0.02 | 1.81 | 1.09 | 7.09 | 81.3 |
| *Pseudoalteromonas spp.* | 0.03 | 0.03 | 1.79 | 1.19 | 7 | 88.3 |

**Figure caption:**

**Fig. S1** Microbial communities are categorized by order in the each site based on the 16SrRNA gene analysis.

**Fig. S2** Putative pathogen composition categorized by genus in the each site based on the 16SrRNA gene analysis.
